# A High Incidence of Sperm with Cytoplasmic Droplets Affects the Response to Bicarbonate in Preserved Boar Semen

**DOI:** 10.3390/ani11092570

**Published:** 2021-08-31

**Authors:** Heiko Henning, Anne-Marie Luther, Dagmar Waberski

**Affiliations:** 1 Unit for Reproductive Medicine, Clinic for Pigs and Small Ruminants, University of Veterinary Medicine Hannover, Foundation, 30559 Hannover, Germany; Heiko.Henning@fli.de (H.H.); anne-marie.luther@tiho-hannover.de (A.-M.L.); 2 Institute of Farm Animal Genetics, Friedrich-Loeffler-Institut, 31535 Neustadt am Rübenberge, Germany

**Keywords:** semen preservation, cytoplasmic droplets, capacitation, calcium influx, boar semen

## Abstract

**Simple Summary:**

The loss of sperm quality during liquid preservation of boar semen may depend on the initial characteristics of the raw semen. Retained cytoplasmic droplets (CD) at the sperm tail caused by incomplete sperm maturation in the epididymis are the most common morphological abnormality in boar semen. The aim of the present study was to examine whether the presence of CD affects the quality of boar sperm during long-term liquid storage. For this, sperm integrity and function were evaluated in semen samples containing different amounts of sperm with CD. It was found that the ability of sperm to perform an essential preparative step for fertilization, named capacitation, was impaired in semen samples rich in CD. Moreover, the enhanced presence of CD caused a higher incidence of sperm destabilization in semen doses preserved for long term (96 h). In contrast, sperm motility and viability were not affected by the presence of CD. The proportion of sperm with CD did not decrease during semen storage in the semen extender. It is concluded that a disturbed maturation of sperm in the epididymis impairs essential steps of fertilization and causes a faster sperm destabilization during in vitro storage.

**Abstract:**

Retained cytoplasmic droplets (CD) are the most frequent sperm abnormality in boar semen. A high incidence of CD is associated with subfertility, but the underlaying reasons are not well understood. The storage of extended semen might augment the adverse effects of CD on essential steps towards fertilization, such as capacitation. The aim of this study was to examine whether the enhanced presence of CD in boar semen influences sperm’s response to the capacitation stimulus bicarbonate during long-term semen storage. Extended semen samples (n = 78) from 13 artificial insemination centers were analyzed using a flow cytometric calcium influx assay. Samples with >15% of CD showed a reduced specific response to bicarbonate and a higher non-specific destabilization after storage for 96 h and subsequent incubation at 38 °C in three variants of Tyrode’s medium (*p* < 0.05). The size of the bicarbonate-responsive sperm population was inversely correlated with the presence of CD-bearing sperm (r = −0.61, *p* < 0.01). Samples with ≤15% and samples with >15% of CD did not differ in motility or viability and acrosome integrity during semen storage. In conclusion, incomplete epididymal sperm maturation impairs the in vitro capacitation ability and promotes sperm destabilization in stored boar semen.

## 1. Introduction

Retained cytoplasmic droplets (CD) are regularly addressed as the most frequent morphological sperm abnormality in boar semen [1,2,3,4]. Breeding companies in several countries use thresholds for the maximal acceptable amount of spermatozoa with CD in boar semen, established between 15% and 30% [5]. The special handling of this sperm abnormality is based on the observation that the higher occurrence of CD-bearing sperm is associated with reduced pregnancy rates, farrowing rates, and litter sizes [2,4,6,7]. The underlying reasons for the impaired fertilizing capacity associated with CD is only partially understood. CD contain residual cytoplasm and flattened, single-membrane and double-membrane vesicles, which are partly horseshoe-shaped [8,9]. The vesicles originate from different parts of the Golgi apparatus and the endoplasmic reticulum [10]. On a molecular level, CD contain an almost full machinery of enzymes of the glycolytic pathway [11], equipment for active translation [12], and antioxidant proteins [13]. In addition, CD contain various hydrolases, which are suggested to stem from lysosomal bodies that have been sequestered into CD during sperm maturation [14,15,16]. The rich content of CD is supposed to play a vital role during epididymal sperm maturation [12,17] but, if not shed upon ejaculation, may interfere with the function of post-ejaculatory sperm. This might especially apply if semen is stored for several days in the liquid state, which is the common practice in artificial breeding of pigs. In freshly extended boar semen, higher incidences of CD seem to hamper initial capacitation events [18]. 

In human sperm, the presence of sperm-bearing CD is positively correlated with the production of reactive oxygen species [19], which likewise could explain the intense lipid peroxidation observed in cytoplasmic droplets of (freshly ejaculated) porcine spermatozoa [20]. The interference of ROS with the capacitation cascade and possible other sperm functions is well described [21,22] and might even culminate after in vitro storage.

There is a lack of knowledge about the role of CD in stored boar semen. Currently, the utilization of boar semen that has been stored for more than three days is intensified to enhance the profitability of semen production in artificial insemination (AI) centers. Concomitantly, tendencies to reduce the number of spermatozoa in the semen dose are becoming more common. To avoid decreases in insemination results, a profound knowledge about the interplay between sperm morphology with a special focus on CD and sperm function is required. 

The aim of this study was to investigate the sperm’s response to the capacitating agent bicarbonate in stored boar semen samples with enhanced amounts of CD. For this, a large-scale experiment using random samples from a semen quality monitoring program was conducted. Moreover, the relationship between the presence of CD and basic sperm quality traits was determined.

## 2. Materials and Methods

### 2.1. Chemicals

All chemicals were obtained from Merck KGaA (Darmstadt, Germany) and Carl Roth GmbH & Co. KG (Karlsruhe, Germany) unless otherwise stated. Propidium iodide (PI) was purchased from Sigma-Aldrich Productions GmbH (Steinheim am Malbuch, Germany). PNA-FITC and Fluo-3/AM were from Axxora Deutschland GmbH (Lörrach, Germany).

### 2.2. Semen Samples

Twenty extended semen samples of from each of 13 AI centers were obtained during a routine quality control monitoring program [23]. Ninety percent of the boars were Piétrain, reflecting the most frequent breed in the participating AI stations. The remaining boar breeds were crossbreeds, Landrace and Large White. Entire ejaculates (sperm-rich and sperm-poor fractions) were collected by the gloved-hand method and processed in accordance with standard procedures of the individual boar stud. The semen extender for all samples was Beltsville Thawing Solution (BTS). Semen samples diluted to 21 ± 9 × 10^6^ sperm/mL were shipped overnight in temperature-controlled boxes (16–18 °C) to the laboratory at the Unit for Reproductive Medicine, University of Veterinary Medicine Hannover, Foundation, Hannover, Germany. Upon arrival, the temperature in transport boxes was controlled, and samples were stored at 17 °C. Samples were first analyzed 24 ± 6 h after semen collection. Six samples from each AI center were chosen to represent the two highest, average, and lowest quality samples with respect to the number of viable spermatozoa with intact acrosome membranes (PI- and PNA-FITC-negative sperm), which resulted in n = 78 samples. 

In addition, 11 extended semen samples (n = 11) with an elevated number of spermatozoa with cytoplasmic droplets were assessed for changes in the percentage of sperm with proximal or distal cytoplasmic droplets after 24 h and 96 h of storage at 17 °C. In addition, cells were also evaluated prior to and after a 120 min incubation period at 38 °C after 24 h and 96 h storage, respectively.

### 2.3. Assessment of Sperm Morphology

Sperm morphology was assessed in 200 spermatozoa from each extended semen sample after liquid fixation of 500 to 1000 µL diluted semen in 300 µL fixating buffer (10 mM citric acid in aqua dest. with 4% formalin (*v*/*v*)) using phase-contrast microscopy (×1000, oil immersion). The fixative was used at room temperature. Spermatozoa were classified according to a simplified scheme based on morphology classification proposed by Krause (1966) [24]. In the case of multiple defects per cell, only the most severe abnormality was considered. The hierarchy for severity of sperm defects was: (1) loose heads, (2) head abnormalities (e.g., tapered head), (3) acrosome abnormalities (e.g., detached acrosome), (4) form abnormalities in the mid-piece, principal, and end piece (e.g., bent tails), and (5) CD.

### 2.4. Computer-Assisted Semen Analysis

Extended semen (4 mL) was incubated for 15 min at 38 °C in a water bath, and motility assessed with the CASA-system SpermVision^®^ (Minitüb GmbH, Tiefenbach, Germany) using four-chamber slides (Leja Products B.V., Nieuw-Vennep, the Netherlands) with a chamber depth of 20 µm. The CASA-system was equipped and operated as described by Henning et al. (2012) [25]. A camera providing 648 by 484 active pixel (AccuPixel TM6760 CL, JAI A/S, Glostrup, Denmark) with a 0.63 camera adapter (U-PMTVC tv-0.63, Olympus Europa SE & Co. KG, Hamburg, Germany) was used. A spermatozoon was classified as motile when its average head orientation change (AOC) exceeded 2.5° and classified as progressively motile when the distance moved from A to B in a straight line (DSL) was longer than 4.5 µm.

### 2.5. Assessment of Plasma Membrane and Acrosome Integrity

The assessment of plasma membrane and acrosome integrity was performed as described by Henning et al. (2012) [25]. Briefly, an aliquot of the diluted semen sample was stained with propidium iodide (PI; final concentration 2.5 µg/mL) and peanut agglutinin conjugated to fluorescein-isothiocyanate (PNA-FITC; final concentration 3.0 µg/mL) for 5 min at room temperature. A subsample of 5 µL was transferred to 895 µL of a HEPES-buffered saline medium (HBS; pH 7.40 ± 0.05 at room temperature, 300 ± 5 mOsmol/kg), and 10,000 events were analyzed with a DAKO ‘Galaxy’ flow cytometer cytometer (Dako Deutschland GmbH, Hamburg, Germany) controlled by ‘FloMax^®^’ software (version 2.4, Sysmex Partec GmbH, Münster, Germany).

### 2.6. Assessment of Responsiveness to Bicarbonate

A simplified protocol for a flow cytometric calcium influx assay [26] was used to assess the specific responsiveness of boar spermatozoa to bicarbonate for 78 semen samples after 24 h and 96 h of storage at 17 °C. For this, an aliquot of diluted semen (4 mL) was incubated with 4 µL of a Fluo-3/AM stock solution (1 mM in dimethylsulfoxide; final concentration: 1 µM) for 30 min at room temperature in the dark. After 15 min of incubation, the cell suspension was mixed again and incubated for a further 15 min. After loading, the sperm suspension was kept in the dark and used within 30 min after preparation.

Aliquots of Fluo-3-loaded spermatozoa were incubated at a concentration of 1 to 1.5 × 10^5^/mL in three variants of Tyrode’s medium. The capacitating variant contained 15 mM bicarbonate and 2 mM calcium (Tyr_BicCa_), whereas the non-capacitating, i.e., bicarbonate- free variants contained either 2 mM calcium (Tyr_Ca_) or 1 mM EGTA (Tyr_Control_). Tyr_BicCa_ consisted of 96 mM NaCl, 20 mM HEPES, 5 mM glucose, 3.1 mM KCl, 0.4 mM MgSO_4_, 0.3 mM KH_2_PO_4_, 100 µg/mL gentamycin sulfate (SERVA Electrophoresis GmbH, Heidelberg, Germany), 20 µg/mL phenol red, 1.0 mM sodium pyruvate, 21.7 mM sodium lactate, 3 mg/mL bovine serum albumin (Cohn’s fraction V, fatty acid free), 15 mM NaHCO_3_ (Bic), and 2 mM CaCl_2_ (Ca). All media were adjusted to a pH of 7.40 ± 0.05 at 38 °C and an osmolality of 300 ± 5 mOsmol/kg. The osmolality of the control media was adapted by increasing the NaCl content. Propidium iodide (PI; final concentration: 2.5 µg/mL) was added before incubation of the spermatozoa started. Tyr_BicCa_ was kept in an incubator under 5% CO_2_ and 100% humidity for equilibration, whereas Tyr_Ca_ and Tyr_Control_ were kept sealed in a heating cabinet.

The samples were analyzed after 3 min and 60 min incubation on a DAKO ‘Galaxy’ flow cytometer (Dako Deutschland GmbH) controlled by ‘FloMax^®^’ software (version 2.4, Sysmex Partec GmbH). HBS medium (pH 7.40 ± 0.05 at 38 °C, 300 ± 5 mOsmol/kg) was used as sheath fluid. The dyes were excited by an argon ion laser (20 mW) at 488 nm. The gating strategy and filter sets were identical to earlier descriptions [25,26]. Signals for PI allowed distinguishing cells with defective (PI-positive, PI^pos^) and intact plasma membrane (PI-negative, PI^neg^). Fluo-3 was used to further subdivide the PI-negative sperm population into cells with a low Fluo-3 fluorescence signal (Fluo-3^low^) and those with a higher Fluo-3-fluorescence signal (Fluo-3^high^). The results were corrected for non-sperm particles according to descriptions by Petrunkina et al. (2010) [27] and Henning et al. (2015) [26]. 

The response of spermatozoa to the incubation conditions was calculated based on the PIneg/Fluo-3neg sperm population. The difference (Δ) between the values at the beginning (3 min) and after 60 min incubation (Δ_3–60 min_ = 3 min–60 min) [26,28] was defined as response in a given medium. The specific response to bicarbonate was calculated as the difference (Δ) in response to Tyr_BicCa_ minus the response to Tyr_Ca_ within 60 min. In analogy, the specific response to calcium was calculated as the difference (Δ) in response to Tyr_Ca_ and Tyr_Control_ within a 60 min incubation period.

### 2.7. Statistical Analysis

Data were analyzed using Excel^®^ and IBM SPSS Statistics^®^ (Version 24; IBM Corp. Armonk, NY, USA). Semen parameters were tested for normal distribution by a Shapiro–Wilk test. Comparisons of semen parameters between samples with a low (≤15%) and a high (>15%) number of spermatozoa with CD were made by a Student’s *t*-test for independent samples. Levene’s test confirmed equality of variances in both groups. Changes in parameters with storage time were analyzed with Wilcoxon’s signed-rank test for paired observations. Correlations were calculated by the Spearman rank correlation coefficient. The likelihood of an increased rate of sperm destabilization in Tyr_Control_ occurring after 96 h storage was estimated with Fisher’s exact test. The significance level was set at *p* < 0.05.

## 3. Results

### 3.1. Semen Parameters after 24 h Storage

**Figure 1:**
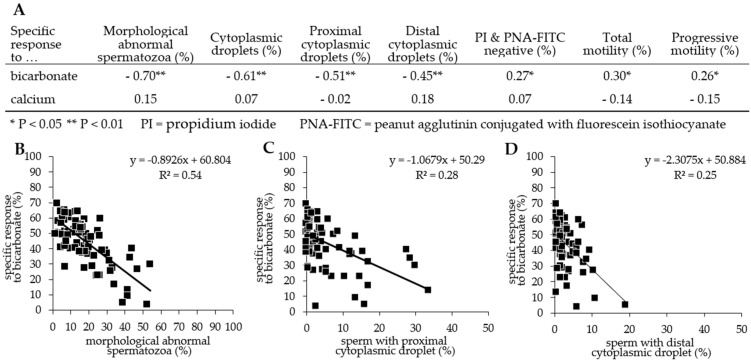
Correlation between semen parameters. (**A**) Spearman rank correlation coefficients for selected sperm parameters measured after 24 h storage at 17 °C (n = 78 boars). The specific response to bicarbonate inversely correlated with (**B**) the percentage of morphological abnormal spermatozoa, (**C**) the percentage of spermatozoa with proximal cytoplasmic droplets, and (**D**) the percentage of spermatozoa with a distal cytoplasmic droplet.

As expected, the pre-selected samples showed a wide spread for standard semen parameters as well as for parameters derived from the calcium influx assay (Table 1). The predominantly observed defect in sperm morphology were CD (Figure 1), although this abnormality was ranked as the lowest in the hierarchy of the scoring system. The responsiveness in Tyr_BicCa_ ranged from 16.5% (minimum: min) to 74.4% (maximum: max). In both control media, the responsiveness ranged from zero to maximal values of 23.0% (Tyr_Ca_) and 17.7% (Tyr_Control_). Based on the responsiveness in the individual media, the specific response to bicarbonate ranged from 3.4% (min) to 69.3% (max), and the specific response to calcium from −4.1% (min) to 13.7% (max).

### 3.2. Correlation between Responsiveness and Standard Semen Parameters

**Figure 2:**
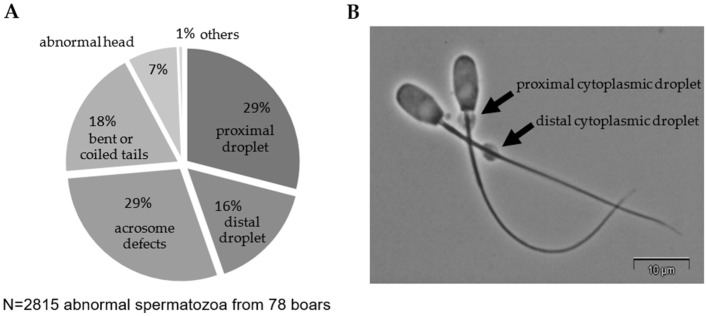
Morphological defects in short-term stored semen samples. (**A**) Distribution of sperm defects in the samples analyzed after 24 h storage at 17 °C. In case of multiple abnormalities per spermatozoon, only one defect was assigned to the cells. The hierarchy for scoring sperm defects was: (1) multiple sperm parts (e.g., two tails), (2) loose heads, (3) acrosome defects (e.g., detached acrosome), (4) head abnormalities (e.g., tapered head), (5) abnormalities in the neck, mid-piece, principal, and end piece (e.g., bent tails), and (6) cytoplasmic droplets. (**B**) Exemplary phase-contrast image of boar spermatozoa with either a proximal or a distal cytoplasmic droplet.

The calcium influx and viability parameters used for the calculation of the responsiveness parameters in different incubation media are shown in Supplementary Table S1. The specific responsiveness to bicarbonate correlated only weakly with the percentage of PI- and PNA-FITC-negative spermatozoa, total motility, or progressive motility (Figure 2). 

On the contrary, the specific responsiveness to bicarbonate was inversely correlated with the amount of morphological abnormal spermatozoa (r = −0.70, *p* < 0.01) and, more specifically, with the amount of spermatozoa with CD (r = −0.61, *p* < 0.01; Figure 2). The correlation was mainly based on the responsiveness of spermatozoa in Tyr_BicCa_, as indicated in Supplementary Table S2. The percentage of morphologically abnormal spermatozoa had a weak inverse correlation with the percentage of viable, acrosome-intact ones, i.e., PI- and PNA-FITC-negative cells (r = −0.30, *p* < 0.01) and no correlation with total or progressive motility. The correlations between the percentage of viable, acrosome-intact spermatozoa and total motility or progressive motility were low (r = 0.36 and 0.33; *p* < 0.01).

### 3.3. Responsiveness to Bicarbonate in Relation to the Occurrence of CD

**Figure 3:**
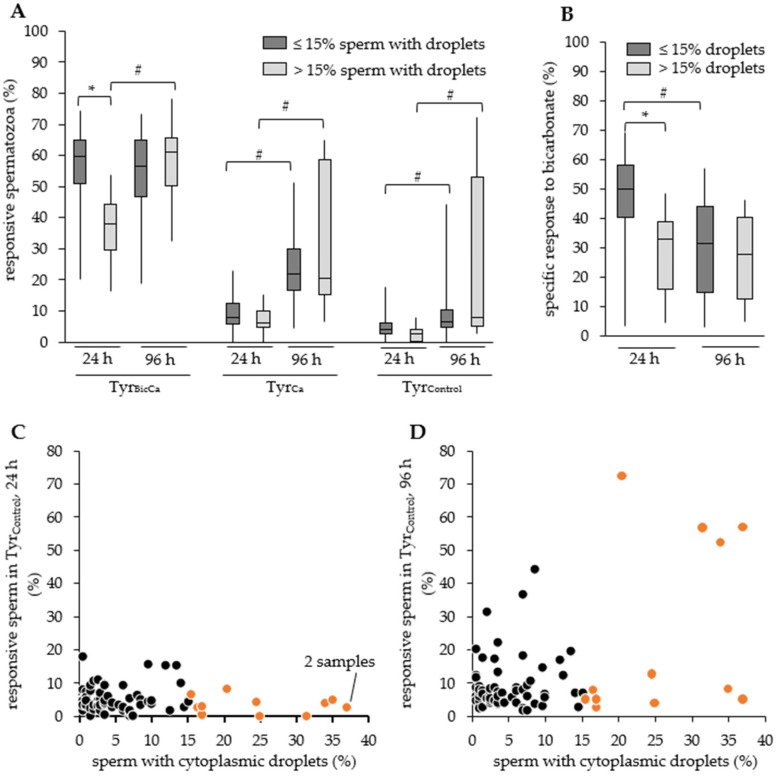
Storage effect on sperm responsiveness to capacitation stimuli in relation to the presence of sperm with cytoplasmic droplets. (**A**) Responsive spermatozoa in different media after 24 h or 96 h semen storage for samples with ≤15% of spermatozoa with cytoplasmic droplets (n = 66 boars) or >15% of spermatozoa with cytoplasmic droplets (n = 12 boars). Responsiveness was calculated based on the propidium iodide- and Fluo-3-low sperm population as the difference (Δ) between the values at the beginning (3 min) and after 60 min incubation (Δ3–60 min = 3 min–60 min) in a capacitating medium with 15 mM bicarbonate and 2 mM calcium (Tyr_BicCa_) or in non-capacitating variants with either 2 mM calcium (Tyr_Ca_) or 1 mM EGTA (Tyr_Control_). * indicates significant differences between the sample groups (*p* < 0.05). # indicates significant difference for a given group between storage times (*p* < 0.05). (**B**) The specific response to bicarbonate was calculated as the difference (Δ) in response to a full capacitating medium (Tyr_BicCa_) within 60 min minus the response to a calcium-containing control medium (Tyr_Ca_) within 60 min. An asterisk indicates significant differences between the sample groups (*p* < 0.05). A hash indicates significant differences for a given group between storage times (*p* < 0.05). (**C**,**D**) Dot plot of the responsive sperm population after 24 h storage (**B**) or 96 h storage (**C**) in relation to the percentage of sperm with cytoplasmic droplets (proximal and distal) within a sample. Orange dots are samples with >15% of spermatozoa with cytoplasmic droplets (n = 12 boars).

Guidelines from the German Livestock Association (BRS) consider only semen samples with ≤15% of spermatozoa with CD as suitable for AI. A comparison of samples with ≤15% of spermatozoa with CD (n = 66; 4.8 ± 0.5%) and samples with >15% of spermatozoa with CD (n = 12; 25.9 ± 2.5%) revealed significant differences in the response to capacitating conditions (Tyr_BicCa_; 56.9 ± 1.4% vs. 36.0 ± 3.5%; *p* < 0.05), but not under control conditions, i.e., in Tyr_Ca_ or Tyr_Control_ (Figure 3A). Consequently, the specific responsiveness to bicarbonate differed between the two groups (47.6 ± 1.6% vs. 28.6 ± 4.1%; *p* < 0.05; Figure 3B). 

**Figure 4:**
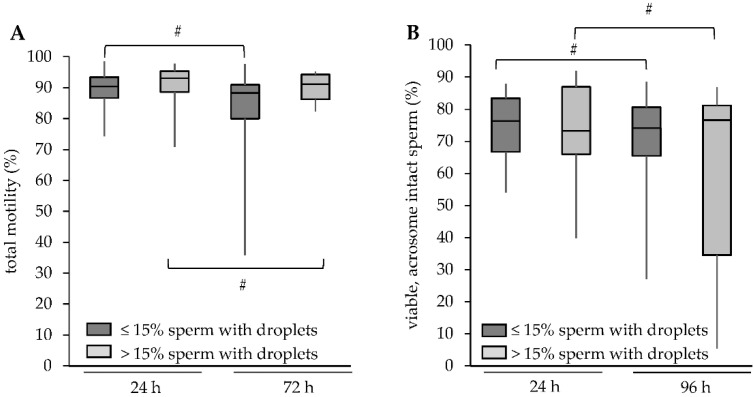
Storage effect on motility and viability in relation to the presence of sperm with cytoplasmic droplets. Change in (**A**) motility and (**B**) viable, acrosome-intact spermatozoa with increasing storage time at 17 °C. A hash (#) indicates significant differences (*p* < 0.05) between the two storage times for samples with ≤15% of spermatozoa with cytoplasmic droplets (CD; n = 66 boars) or samples with >15% of spermatozoa with cytoplasmic droplets (n = 12 boars). A hash indicates significant differences between storage times (*p* < 0.05).

Noteworthy, the groups did not differ with respect to total motility (89.7 ± 5.1% vs. 90.8 ± 7.6%), progressive motility (85.3 ± 8.0% vs. 85.3 ± 14.7%), or the percentage PI- and PNA-FITC-negative spermatozoa (75.1 ± 9.3% vs. 73.4 ± 15.5%; all *p* > 0.05; Figure 4). A small, albeit significant decline during storage in both parameters was evident for samples with ≤15% and >15% of spermatozoa with CD (Figure 4). 

### 3.4. Responsiveness to Bicarbonate in Relation to Storage Duration

Storage of normospermic semen samples, i.e., samples with ≤15% of spermatozoa with CD, resulted in a sperm population that became increasingly responsive to the incubation conditions in bicarbonate-free media (Figure 3A). As a consequence, the sperm population which responded specifically to bicarbonate was significantly reduced after 96 h compared to 24 h of storage (*p* < 0.05; Figure 3B).

The responsive sperm population was also significantly higher in all media for 96 h-stored samples that contained >15% of spermatozoa with CD (Figure 3A), but the number of spermatozoa with a specific response to bicarbonate did not change (*p* > 0.05; Figure 3B). After a 96 h storage period, the sperm population that specifically responded to bicarbonate did not differ between samples containing ≤15% of spermatozoa with CD (30.3 ± 15.5%) and those containing >15% of spermatozoa with CD (26.2 ± 15.2%; *p* > 0.05). 

Samples containing >15% of spermatozoa with CD showed a high variability in the responsiveness of sperm in Tyr_Ca_ and Tyr_Control_ after 96 h storage. This parameter is indicative of a non-specific destabilization in part of the sperm population. After 24 h, none of the samples showed a responsive sperm population of >20% in Tyr_Control_ (Figure 3C). An increased responsive sperm population of >20% after 96 h storage was more likely for samples containing >15% of spermatozoa with CD (4 of 12 samples) than for samples containing ≤15% of spermatozoa with CD (5 of 66 samples; Figure 3D; χ^2^(1) = 6.6, *p* = 0.028).

### 3.5. Change in Cytoplasmic Droplet-Bearing Spermatozoa during Storage or Incubation

**Figure 5:**
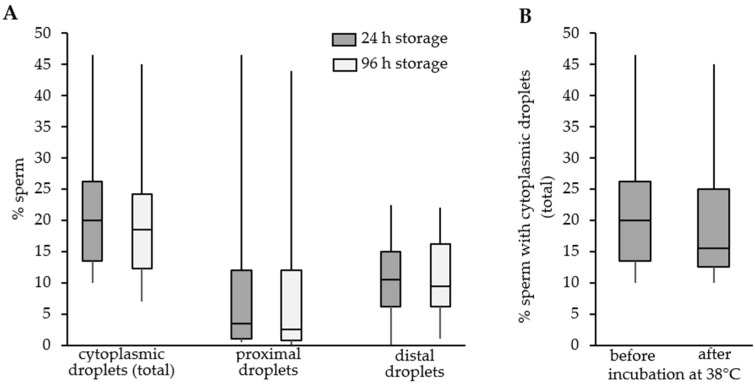
Change in the percentage of sperm with cytoplasmic droplets during storage at 17 °C and subsequent incubation at 38 °C. Semen samples from individual boars (n = 11) were scored for the presence of spermatozoa with proximal or distal cytoplasmic droplets. (**A**) Two hundred cells were evaluated after 24 h and 96 h storage at 17 °C in the BTS extender. Cells were fixated in fixating buffer prior to evaluation. (**B**) Similarly, cells were evaluated after 24 h storage prior to or after 120 min incubation at 38 °C. No significant difference was observed due to storage or incubation time (*p* > 0.05). Incubation at 38 °C after 96 h storage had also no effect (data not shown).

Storage of diluted semen samples at 17 °C had no effect on the percentage of spermatozoa with either proximal or distal CD (Figure 5A; *p* > 0.05). Likewise, incubation of semen samples for 120 min at 38 °C after 24 h (Figure 5B) or 96 h (data not shown) had no impact on the percentage of spermatozoa with cytoplasmic droplets (*p* > 0.05). 

## 4. Discussion

The present data revealed that an enhanced incidence of CD reduces the responsiveness of boar semen to the capacitating stimulus bicarbonate and increases non-specific destabilization after long-term storage in a subset of semen samples. The results are in line with the observation that samples with a higher incidence of CD show increased membrane fluidity after stimulation with bicarbonate [18]. In the cited study, the lack of response was attributed to incomplete reduction in cholesterol in the plasma membrane during epididymal transit. In the present study, the response to bicarbonate was assessed by calcium influx, which is a later step in the signal cascade of capacitation. It remains unknown, however, whether it is indeed the spermatozoa with a CD that do not respond, because tracking of single spermatozoa was not carried out. The presence of CD in ejaculated sperm is commonly regarded as an indication of incomplete sperm maturation during transit through the epididymis. Considering that sufficient epididymal maturation of spermatozoa seems to be a prerequisite for gaining full functional competence in preparation for fertilization [29], a direct association between the presence of CD and a reduced response to a capacitation stimulus can be suggested.

Our representative data using semen doses from 13 AI centers confirm previous experimental data [30] that show a loss of the in vitro capacitation ability of semen samples stored for 96 h in the BTS extender. Here, we demonstrate that the presence of more than 15% of CD-bearing spermatozoa in long-stored semen was associated with a higher risk for viable sperm to show instability during incubation at 38 °C in the absence of bicarbonate as assessed by increased cytosolic calcium levels. Noteworthy, CD are rich in intracellular calcium [31], and many of the vesicular structures in CD of ejaculated human sperm are positive for the inositol 1,4,5-trisphosphate receptor (IP3R) and calreticulin [32]. In analogy to the function of these calcium-regulating structures in somatic cells [33,34], calcium release from remnants of the endoplasmic reticulum in retained CD of boar spermatozoa may contribute to the increase in cytosolic calcium. Regardless of the underlying mechanism, an increase in intracellular calcium contributed to the bicarbonate-independent destabilization of spermatozoa in our experiments, which were characterized by a high responsiveness and increased rate of cell death in Tyr_Ca_ and Tyr_Control_ and low values for viable, acrosome-intact sperm.

Our in vitro observation strengthens earlier perceptions from in vivo trials showing that an elevated percentage of spermatozoa with a retained CD is a risk factor for subfertility, especially when semen samples are used after prolonged storage [4].

Noteworthy, in raw semen, a reduction in boar sperm with retained CD from 18.7% to 2.8% during 48 h semen storage at 15 °C was reported [35]. In contrast, exposure of ejaculated boar sperm to uterine fluid for up to 24 h at 38 °C did not reduce the incidence of CD [36]. Likewise, in the present study, exposure to the semen extender with only low amounts (5 to 10% (*v*/*v*) seminal plasma) during storage at 17 °C for 96 h and subsequent thermic stress (120 min, 38 °C) did not reduce the presence of sperm with retained CD. Whether long-term semen storage leads to a progressive degradation of CD and concomitant release of enzymes and ROS causing loss of sperm function remains to be shown.

Retained CD are regularly addressed as the most frequent sperm abnormality [1,2,3,4,37], which agrees with our current dataset. Our results probably even underestimate the true incidence because CD were in the lowest position of the counting hierarchy. Thus, a CD is not scored as defect when another defect is simultaneously present in the same spermatozoon. Moreover, in AI practice, a morphological analysis of raw semen is too time-consuming to be considered prior to semen processing. Currently available CASA systems allow for automated real-time detection of CD (and bent tails) in one step with motility assessment without inference of other morphological abnormalities. Since our data revealed no difference in motility and viability of semen samples with low or high CD incidence, and only low correlations between motility and viability to the bicarbonate responsiveness were found, the additional consideration of CD by CASA analysis will be a valuable additional criterion when selecting ejaculates for use in AI.

## 5. Conclusions

Incomplete epididymal sperm maturation reduces the in vitro capacitation ability and promotes destabilization of boar spermatozoa during in vitro storage. This emphasizes the importance of selecting ejaculates at the level of semen processing for the number of sperm with CD in order to provide AI doses with a high chance of capacitation-competent spermatozoa.

## Figures and Tables

**Table 1:** Quality parameters for semen samples after 24 h storage at 17 °C. Semen samples had been stored in Beltsville Thawing Solution (n = 78 boars). The responsiveness of sperm in the calcium influx assay was calculated based on the propidium iodide and Fluo-3 low sperm population as the difference (Δ) between the values at the beginning (3 min) and after 60 min of incubation (Δ3–60 min = 3 min–60 min). The specific response to bicarbonate was calculated as the difference (Δ) in response to a full capacitating medium (Tyr_BicCa_) within 60 min minus the response to a calcium-containing control medium (Tyr_Ca_) within 60 min. The specific response to calcium was calculated as the difference (Δ) in response to a full capacitating medium (Tyr_BicCa_) minus the response to a calcium-free control medium (Tyr_Control_).

Parameter	Mean	sd	Minimum	Maximum
Total motility (%)	89.9	5.5	70.9	98.4
Progressive motility (%)	85.3	9.2	42.4	96.2
VAP (µm/s)	66.5	11.7	25.0	94.2
VCL (µm/s)	117.9	23.8	43.8	166.4
VSL (µm/s)	51.3	9.2	19.7	80.9
ALH (µm)	3.11	0.70	1.04	5.02
BCF (Hz)	33.5	4.8	17.6	42.2
PI and PNA-FITC negative sperm (%)	74.8	10.4	39.8	91.9
Morphological abnormal sperm (%)	18.0	12.4	1.5	54.0
Cytoplasmic droplets (%)	8.1	9.1	0.5	37.0
Proximal cytoplasmic droplets (%)	5.7	7.6	0.0	33.5
Distal cytoplasmic droplets (%)	3.3	3.3	0.0	19.0
Response in Tyr_BicCa_	53.7	13.7	16.5	74.4
Response in Tyr_Ca_	9.0	5.2	0.0	23.0
Response in Tyr_Control_	4.6	3.9	0.0	17.7
Specific response to bicarbonate	44.7	15.1	3.4	69.3
Specific response to calcium	4.4	4.1	−4.1	13.7

Tyr_BicCa_ = Tyrode’s medium with 15 mM bicarbonate and 2 mM Ca^2+^; Tyr_Ca_ = Tyrode’s medium without bicarbonate, with 2 mM Ca^2+^; Tyr_Control_ = Tyrode’s medium without bicarbonate, with 1 mM EGTA; PI = propidium iodide; PNA-FITC = fluorescein isothiocyanate-conjugated peanut agglutinin; VAP = average path velocity; VCL = curvilinear velocity; VSL = straight-line velocity; ALH = amplitude of lateral head displacement; BCF = beat cross frequency.

## Data Availability

The data presented in this study are available on request from the corresponding author.

## Supplementary Materials

The following are available online at https://www.mdpi.com/article/10.3390/ani11092570/s1, Table S1: Cell populations from the calcium influx assay, Table S2: Spearman rank correlation coefficients.
