# Supplementary material for: A High Incidence of Sperm with Cytoplasmic Droplets Affects the Response to Bicarbonate in Preserved Boar Semen
Source: Animals (Basel). 2021 Aug 31;11(9):2570. doi: 10.3390/ani11092570 (PMC8465936; doi:10.3390/ani11092570)
Supplement: Supplementary file 1 [file animals-11-02570-s001.zip › animals-1357604-supplementary/Supplemental Table 1_R.pdf]

A

		Viable, low calcium		Viable, high calcium		Non-viable	
		≤ 15 % droplets*	> 15 % droplets**	≤ 15 % droplets*	> 15 % droplets**	≤ 15 % droplets*	> 15 % droplets**
Tyr_{BicCa}	3 min	81.4 ± 6.7	83.3 ± 3.7	6.7 ± 3.3	5.7 ± 1.8	11.9 ± 5.0	11.0 ± 3.2
	60 min	24.3 ± 11.7 ^{a}	47.4 ± 12.3 ^{b}	29.7 ± 8.7 ^{a}	20.1 ± 7.6 ^{b}	45.9 ± 11.1 ^{a}	32.5 ± 8.2 ^{b}
Tyr_{Ca}	3 min	81.2 ± 6.8	83.3 ± 4.6	6.8 ± 3.5	5.8 ± 2.0	12.0 ± 4.8	10.9 ± 3.6
	60 min	71.9 ± 10.9	75.9 ± 8.1	12.0 ± 6.6	8.9 ± 4.4	16.1 ± 7.3	15.2 ± 5.6
$Tyr_{Control}$	3 min	85.2 ± 5.7	86.5 ± 3.4	2.9 ± 2.0	2.0 ± 0.7	11.9 ± 5.0	11.5 ± 3.1
	60 min	80.3 ± 7.7	83.5 ± 5.6	4.2 ± 2.9	2.9 ± 2.0	15.5 ± 6.6	13.6 ± 4.5

* n=66 boars, ** n=12 boars

B

		Viable, low calcium		Viable, high calcium		Non-viable	
		≤ 15 % droplets*	> 15 % droplets**	≤ 15 % droplets*	> 15 % droplets**	≤ 15 % droplets*	> 15 % droplets**
Tyr_{BicCa}	3 min	70.9 ± 14.9 ^{a}	80.2 ± 5.5 ^{b}	13.4 ± 7.6 ^{a}	8.0 ± 2.9 ^{b}	15.8 ± 9.0	11.7 ± 3.4
	60 min	16.8 ± 10.5	22.4 ± 13.8	27.5 ± 8.9	23.1 ± 5.3	55.6 ± 11.6	54.4 ± 13.2
Tyr_{Ca}	3 min	72.5 ± 16.2 ^{a}	80.9 ± 6.3 ^{b}	12.5 ± 8.4	7.7 ± 3.4	14.9 ± 9.5	11.4 ± 3.8
	60 min	48.8 ± 20.2	49.3 ± 25.2	22.9 ± 10.3	17.9 ± 5.6	28.2 ± 16.1	32.8 ± 21.8
$Tyr_{Control}$	3 min	79.8 ± 9.6 ^{a}	85.5 ± 4.7 ^{b}	5.3 ± 3.1 ^{a}	3.0 ± 1.1 ^{b}	15.0 ± 9.0	11.5 ± 4.1
	60 min	70.6 ± 14.4	61.5 ± 27.9	6.7 ± 3.4	7.1 ± 4.0	22.6 ± 13.9	31.4 ± 24.3

* n = 66 boars, ** n = 12 boars

Supplemental Table 1

Cell populations from the calcium influx assay.

A + B) Sperm populations in different media after A) 24 hours or B) 96 hours semen storage for samples with ≤ 15 % spermatozoa with cytoplasmic droplets (n = 66 boars) or > 15 % spermatozoa with cytoplasmic droplets (n = 12 boars). Spermatozoa were incubated in either a capacitating medium with 15 mM bicarbonate and 2 mM calcium (Tyr_{BicCa}) or non-capacitating variants with either 2 mM calcium (Tyr_{Ca}) or 1 mM EGTA ($Tyr_{Control}$). Cells were identified based on propidium iodide staining as either non-viable (PI-positive) or viable (PI-negative). Fluo-3 was used to further subdivide the viable sperm population in cells with a low Fluo-3 fluorescence intensity (=low free intracellular calcium concentration) and cells with a higher Fluo-3-fluorescence intensity (= high free intracellular calcium concentration). Different small letters (a-b) indicate significant differences between samples with ≤ 15 % or > 15 % spermatozoa with cytoplasmic droplets ($P < 0.05$).
