# Supplementary material for: A High Incidence of Sperm with Cytoplasmic Droplets Affects the Response to Bicarbonate in Preserved Boar Semen
Source: Animals (Basel). 2021 Aug 31;11(9):2570. doi: 10.3390/ani11092570 (PMC8465936; doi:10.3390/ani11092570)
Supplement: Supplementary file 1 [file animals-11-02570-s001.zip › animals-1357604-supplementary/Supplemental Table 2_R.pdf]

Supplemental Table 2

Spearman rank correlation coefficients for selected sperm parameters measured after 24 hours storage at 17 °C (n = 78).

Response in medium…	Morphological abnormal spermatozoa (%)	Proximal & distal cytoplasmic droplets (%)	Proximal cytoplasmic droplets (%)	Distal cytoplasmic droplets (%)	PI & PNA-FITC negative (%)	Total motility (%)	Progressive motility (%)
Tyr_{BicCa}	-0.74**	-0.69**	-0.58**	-0.53**	0.20	0.17	0.13
Tyr_{Ca}	0.07	-0.07	-0.10	-0.05	-0.24	-0.44**	-0.35**
$Tyr_{Control}$	-0.07	-0.15	-0.12	-0.15	-0.42**	-0.31**	-0.21

** P < 0.01 PI = propidium iodide PNA-FITC = peanut agglutinin conjugated with fluorescein isothiocyanate
